# Natural HCV variants with increased replicative fitness due to NS3 helicase mutations in the C-terminal helix α_18_

**DOI:** 10.1038/srep19526

**Published:** 2016-01-20

**Authors:** Claudia Stross, Tetsuro Shimakami, Katrin Haselow, Monazza Q. Ahmad, Stefan Zeuzem, Christian M. Lange, Christoph Welsch

**Affiliations:** 1 Department of Internal Medicine 1, Goethe University Hospital Frankfurt, 60590 Frankfurt a.M., Germany; 2 Department of Gastroenterology, Kanazawa University Hospital, Kanazawa, Japan

## Abstract

High replicative fitness is a general determinant of a multidrug resistance phenotype and may explain lower sensitivity to direct-acting antiviral agents (DAAs) in some hepatitis C virus genotypes. Genetic diversity in the molecular target site of peptidomimetic NS3 protease inhibitors could impact variant replicative fitness and potentially add to virologic treatment failure. We selected NS3 helicase residues near the protease natural substrate in the NS3 domain interface and identified natural variants from a public database. Sequence diversity among different genotypes was identified and subsequently analyzed for potential effects of helicase variants on protein structure and function, and phenotypic effects on RNA replication and DAA resistance. We found increased replicative fitness in particular for amino acid substitutions at the NS3 helicase C-terminal helix α_18_. A network of strongly coupled residue pairs is identified. Helix α_18_ is part of this regulatory network and connects several NS3 functional elements involved in RNA replication. Among all genotypes we found distinct sequence diversity at helix α_18_ in particular for the most difficult-to-treat genotype 3. Our data suggest sequence diversity with implications for virus replicative fitness due to natural variants in helicase helix α_18_.

Peptidomimetic protease inhibitors (PIs) are key direct-acting antiviral agents (DAAs) against the hepatitis C virus (HCV) designed to target the catalytic site of the genotype 1 virus NS3 protease domain (NS3p)^1^. They mimic the natural protease substrate to inhibit polyprotein processing as a central step in viral RNA replication. Although the protease activity of NS3 has been the focus of drug development efforts, NS3 is a bifunctional enzyme with a separate serine protease and a DExD-box RNA helicase domain connected by a flexible linker. The domain interface sustains the NS3p catalytic site, which is the target site for PIs. The helicase domain (NS3h) has NTPase and 3′–5′ RNA unwinding activity, which is essential for HCV RNA synthesis^1^. An allosteric pocket in the domain interface regulates conformational changes between an open and closed conformation in the full-length NS3 protein which is pivotal for HCV replicase formation^2^. The N-terminal 21 amino acids of NS4A form a transmembrane α-helix that together with NS3p helix α_0_ is required for integral membrane association of the protein complex^3^. The attachment of the two domains in the full-length NS3 protein has been shown to strongly influence their individual properties. The linker region connecting the two domains is not required for enzymatic activity but seems critical for replication and infectivity either in modulating conformation of full-length NS3 or in mediating interactions between NS3 and other viral or host proteins during the HCV life cycle. Binding of PIs to the active site of NS3p blocks processing of the viral polyprotein and hence RNA synthesis but also impacts a late stage in virus assembly/maturation^4^. The development of chronic hepatitis C depends largely on the capability of NS3p to cleave antiviral host proteins. A prominent example is the cleavage (and inactivation) of mitochondrial antiviral-signaling protein MAVS (also called Cardif, VISA, and IPS-1), which impedes MAVS-mediated induction of type I interferons by the retinoic acid-inducible gene I (RIG-I) signaling pathway^5^,^6^.

In the typical HCV infected patient, the error-prone viral replication machinery results in a genetically diverse virus population, so-called quasi-species, with most, if not all single and double mutants likely to pre-exist at low frequencies in the cloud of viral variants^7^. Related to the HCV mutational rate and the virus capability to tolerate NS3 sequence variations, PIs against NS3 are burdened with a particularly high risk for development of resistance-associated amino acid variants (RAVs)^8^. The genetic diversity is closely linked to molecular mechanisms related to variant escape from PI pressure where HCV genotype-specific response rates in antiviral treatment are shown to correlate with the nature and natural frequency of mutations^9^,^10^.

RAVs display reduced drug susceptibilities, however, due to large scale conformational changes between the open and closed conformation of NS3, helicase mutants are unlikely to have a direct impact on peptidomimetic PI binding to the protease active site^2^. Importantly, RAVs in the absence of drug pressure generally have a lower fitness than the wild type, owing to a mutation-incurred cost, which is likely to determine which variants emerge as dominant from a quasi-species population^8^. Thereby, Sheldon *et al.* recently showed that replicative fitness by itself is a potent mechanism of multidrug resistance in chronic HCV infection. They demonstrated that cross-resistance among antiviral inhibitors may come about not only through RAVs that confer resistance to more than one drug but also through a general increase in viral fitness. High level replicative fitness allows HCV to maintain mechanisms to shutoff host cell protein synthesis by multiple viral expression products that interact with host factors. Moreover, high levels of replicating viral RNA and RNA expression products may confer a selective advantage in competition with the number of inhibitor molecules that reach the target viral or cellular proteins at the replication complex^11^.

To date, genotype 3 has become the most difficult-to-treat with DAA-based regimens and displays a specific clinical phenotype with more rapid progression of liver disease and a higher incidence of hepatocellular carcinoma^12^,^13^. However, we are still far from an understanding of the molecular mechanisms underlying genotype-specific response rates, although these clinical observations might relate to molecular mechanisms in viral replication that compensate for viral variant fitness deficits^8^. Differences in the genetic amino acid variation (diversity) in NS3 between HCV genotypes might explain part of the genotype-specific response reported for antiviral treatment with PIs. Since residues from both NS3 domains interact with the peptide substrate of the NS3 protease, NS3h domain-interface polymorphisms could potentially contribute to treatment response by modulating replicative fitness. In the present study, we have analyzed the natural pre-treatment variation in NS3h amino acid residues that neighbor the natural substrate of proteolysis in the NS3 domain interface from comprehensive sets of genotype 1a, 1b, 2 and 3 dominant strains deposited in a public database. Respective amino acid substitutions were introduced into a subgenomic replicon system to determine their impact on susceptibility to peptidomimetic PIs and virus replicative fitness.

## Results

### Strategy and mutants

We used the protease-helicase complex structure PDB 1CU1 to identify amino acid residues in NS3h within close structure proximity to the natural substrate of NS3p-mediated proteolysis. Eighteen residues neighboring the NS3p substrate were identified: residues 438, 488, 524–532, 534, 619–624 (Table 1, Supplementary Fig. S1), subsequently denoted NS3h domain-interface residues. For those residues we identified differences from the consensus sequence in genotype 1a, 1b, 2 and 3 sequences downloaded from euHCVdb. Importantly, none of the patients from which these sequences were derived appear to have been treated previously with a DAA (see Materials and Methods). In genotype 1a and 2 we found only 5 of the NS3h domain-interface residues with polymorphisms, whereas genotype 1b showed polymorphisms at 14 residues and genotype 3 at 7 residues.

Importantly, some of the variants were observed more frequently in the database than others. Most abundant variants in genotype 1b were S534G, A621T and S624A that were identified 74, 35 and 10 times respectively. Some variants were observed only once in the database, including F438L, Q526M, D527V, H528Y, S534A, S534D, C622S and S624W. A comparison of the sequence diversity for all genotypes is given in Table 1. Based on this analysis, we were choosing the genotype with highest sequence diversity, genotype 1b, and the respective Con1 replicon system for phenotype characterization of natural variants. We created 13 mutants in the backbone of the Con1 replicon coding for the enzyme variants 438 L, 524 F/I, 525 W, 526 M, 527 V, 528Y, 532 S, 534 G, 619 V, 620 T, 621 T and 624 A, that were subsequently tested for their impact on RNA replication and resistance against NS3 protease inhibitors.

### Impact of NS3h domain-interface mutations on RNA replication

**Figure 1:**
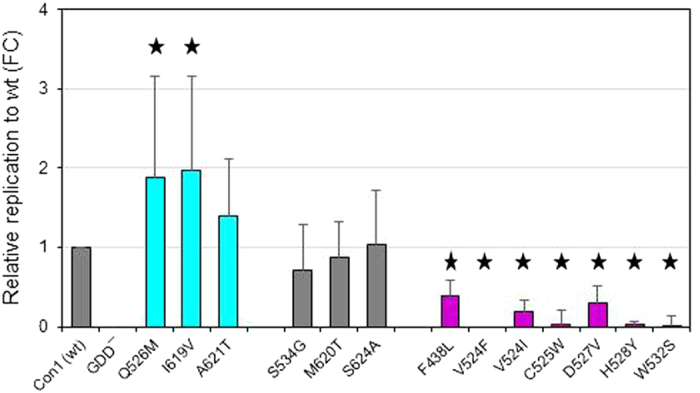
Impact of helicase mutations on HCV RNA replication. HCV RNA determined by two-step reverse transcription quantitative real-time PCR (RT-PCR), normalized to that of the wild type Con1 RNA from each helicase mutant. Bars are color coded as follows: cyan – increased replicative fitness; grey – not different from wild type; magenta – decreased replicative fitness. The data shown represent the mean ± SD from at least ten independent experiments. “wt” refers to wild type. Y axis shows relative replication to wt as fold change (FC). An asterisk designates mutants for which the fold change compared to wt is significant by Student t-test (P < 0.05).

The replicative fitness of NS3h domain-interface mutants was assessed by quantitative RNA measurement of Huh7.5 cells harboring respective Con1 replicons. Our data demonstrate that NS3h polymorphisms can have a modulatory effect on RNA replication but do not generally have a consistent effect. We found three mutants (Q526M, I619V, A621T) with a positive effect on RNA replication and seven mutants (F438L, V524F, V524I, C525W, D527V, H528Y, W532S) with a negative effect on RNA replication, whereas three mutants (S534G, M620T, S624A) showed no effect on replicative fitness (Fig. 1, Supplementary Table S1). We did not observe an association between conservative and non-conservative substitutions or physicochemical properties and replicative fitness, but a consistent effect and association between replicative fitness and the position of a mutant residue within the NS3 domain interface was observed (see below). A correlation between the abundance of a natural variant in the database and its replicative fitness is observed. Most variants with replicative fitness higher or equal to that of the wild type Con1 replicon strain were observed multiple times in the database. The only exception is Q526M that was observed once in the database (database entry EU256082), but showed significantly higher replicative fitness than the Con1 wild type (Fig. 1).

### Protein structure context

**Figure 2:**
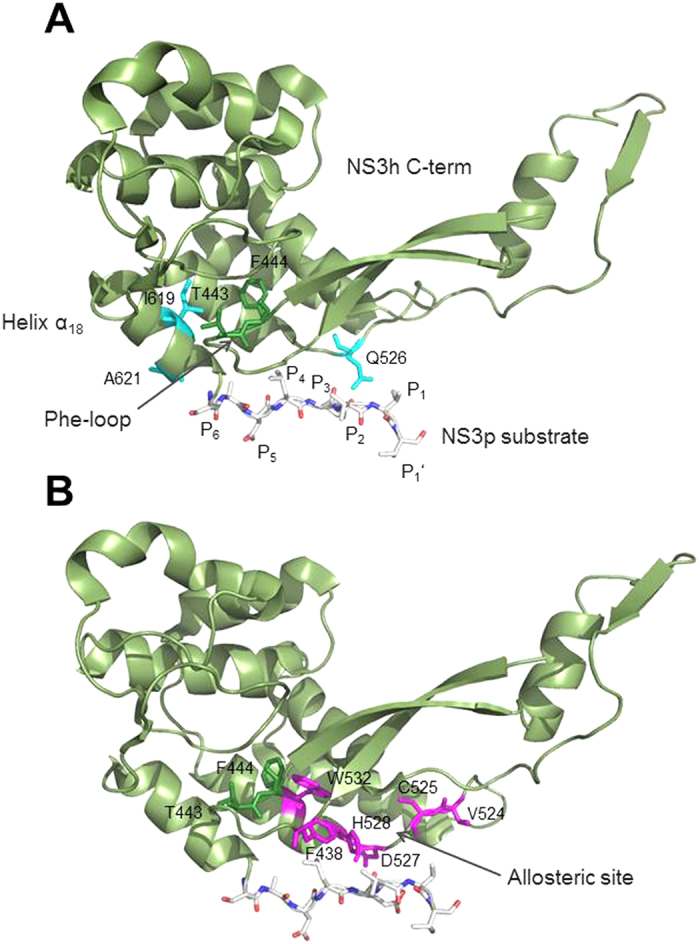
Protein structure context of natural helicase variants. Details of PDB structure 1CU1 showing the C-terminal region of the NS3 helicase (amino acid residues 241 – 631). The NS3 protease natural substrate of proteolysis is given as a stick model with residues denoted as P_6_-P_5_-P_4_-P_3_-P_2_-P_1_-P_1_’ according to the nomenclature of Schechter and Berger^32^, in which residues surrounding a cleavage site are designated from the N- to the C-terminus with the scissile bond located between P_1_ and P_1_’. **(A)** Residues highlighted in cyan where mutations showed increased replication capacity. **(B)** Residues highlighted in magenta where mutations showed decreased replication capacity. Phe-loop residues T443 and F444 are highlighted in green.

Some regulatory residues were located at the domain interface allosteric site that is V524, C525, Q526 and D527. Here a positive effect on RNA replication is observed only for Q526M with its side chain pointing towards the NS3p natural substrate residue P_3_ (E628) (Fig. 2A). All other allosteric site mutations had a negative effect on RNA replication, in particular V524F/I and C525W, with their side chains oriented away from the NS3p natural substrate. H528Y with a negative effect on RNA replication is likely to interact directly with the NS3p peptide substrate at its P_3_ site (E628). A positive effect on RNA replication is observed for I619V and A621T, both located in NS3h C-terminal helix α_18_ (that is P614 – C622, according to PDB 1CU1) preceding the natural substrate of proteolysis. I619 is oriented towards the protein interior with its side chain close to the functionally important Phe-loop residues T443 and F444, whereas A621 is surface exposed and close to the NS3p peptide substrate P_6_ site (A625) (Fig. 2B). A negative effect on replicative fitness is observed for the mutational site W532 that is located at NS3h helix α_14_ (that is L529 – T538, according to PDB 1CU1). Helix α_14_ is oriented anti-parallel to helix α_18_ in the protein structure. The wild-type residue W532 is involved in a T-shaped π-π stacking interaction with the aromatic ring of residue F444. Mapping the comprehensive panel of mutational sites on the 3D protein structure of NS3, we found two functionally important sites with modulating effect on RNA replication, the NS3 protease-helicase allosteric site and NS3h C-terminal helix α_18_. Increased replicative fitness is found in particular for mutations at or close to helix α_18_.

**Figure 3:**
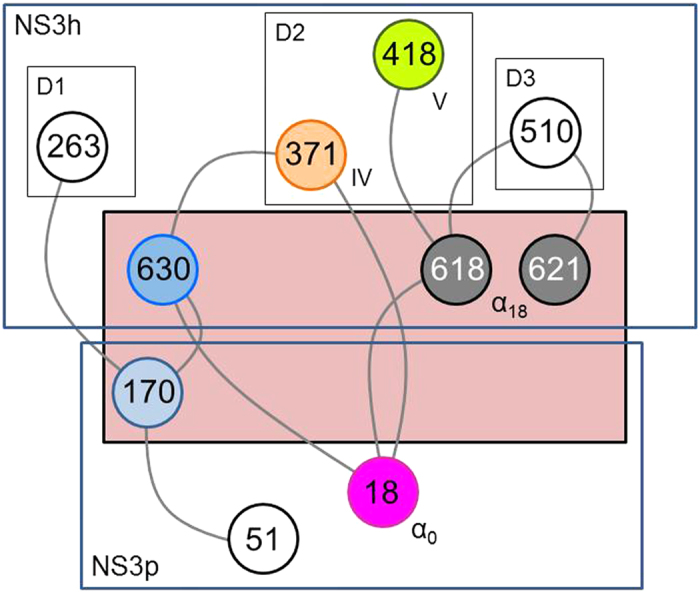
Schematic representation of NS3 coupled residue pairs. Network of NS3 residues (nodes) with correlated mutations. The inter-domain interface is highlighted in salmon. Nodes are color coded to differentiate functional elements and highlight their role in NS3 protein structure and function^33^,^34^: magenta, light blue – peptide and MAVS cleavage; blue – natural protease substrate; grey, orange, green – molecular rearrangements and RNA replication. Annotation: D1 – helicase domain 1; D2 – helicase domain 2; D3 – helicase domain 3; IV – motif IV; V – motif V; α_0_ – helix α_0_; α_18_ – helix α_18_.

Some of the natural NS3h domain-interface polymorphisms we investigated showed very low replicative fitness when introduced into the Con1 backbone. To identify potential molecular mechanisms to compensate for deficits in RNA replication, we performed a correlated mutation analysis in full-length NS3. It can be seen in Table 2 that different networks of coupled residues are found to exist in NS3. A schematic network representation for coupled residue pairs and their functional annotation is given in Fig. 3. A strong coupling between NS3h residues close to the NS3p-substrate of proteolysis and both NS3 domains is observed. We identified 14 coupled residue pairs with an interconnected network of 10 coupled residue pairs, comprising residues of the NS3p peptide substrate, the NS3h C-terminal helix α_18_, as well as helicase and protease functional sites. Six residue pairs consist of NS3h residues, one pair consist of NS3p residues and five pairs connect NS3p with NS3h residues (Table 2). Among those residue pairs we found functionally important sites in NS3h and NS3p. NS3h motif IV, which is involved in intramolecular rearrangements and RNA interaction, is coupled via K371 to the NS3p substrate site P_1_ at V630 and residue I18 of NS3p helix α_0_, which is required for MAVS cleavage and RIG-I pathway control, which, in turn, is coupled to NS3h C-terminal residues Y618 in helix α_18_ and protease substrate site P_1_ at V630. Hence residues I18, K371 and V630 are forming a closed network. Residue I170, which is involved in MAVS binding, is again coupled to V51 in the NS3p domain, which is close to the protease catalytic site. Furthermore, I170 is coupled to the NS3h domain 2 residue A263 and NS3p substrate site P_1_ at V630. Hence there is a strong coupling of two subnetworks with distinct functional sites via NS3p substrate site P_1_. In principle, coupling between those residues may reflect interactions in the denaturated state of NS3, long-range electrostatic interactions or propagation of conformational changes through the protein^14^,^15^. The fact that many of those coupled residues are not charged and the subnetworks involve both NS3 domains, which do not interact with each other in the denaturate state, suggest that the coupling we observed in NS3 is attributable to conformational changes and may reflect pathways of allosteric communication.

### Genotype-specific sequence diversity

**Figure 4:**
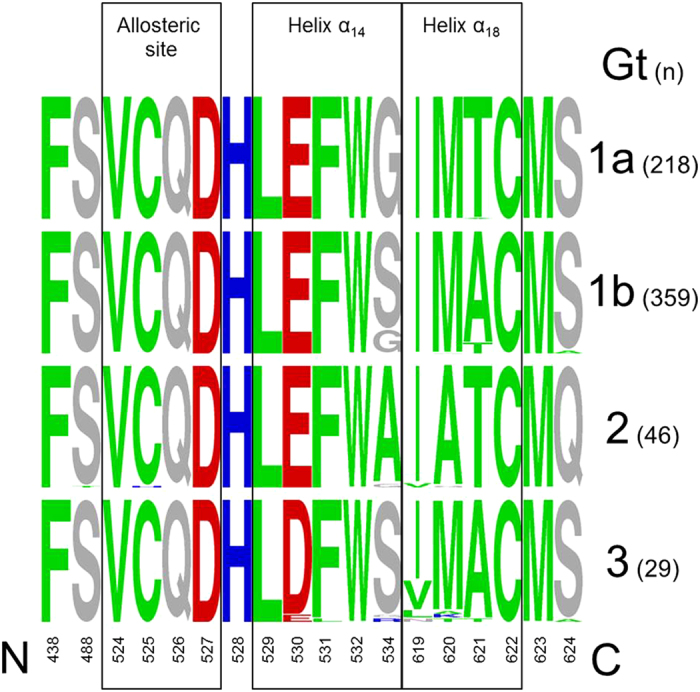
Sequence logo depiction of amino acid sequence diversity within helicase domain-interface residues. The height of each single-character amino acid code is proportional to the representation of that amino acid at each position. The genotype/subtype is indicated on the right with the respective number of sequences downloaded from euHCVdb in brackets. Amino acids are colored as follows, according to their physico-chemical properties: hydrophobic (green) – ACFILMPTVWY; charged, positive (blue) – HKR; charged, negative (red) – DE; rest (grey) – GNQS.

Sequence diversity is likely to reflect variant replicative fitness and adaptive potential and hence potentially provides a molecular determinant for genotype-specific response rates on DAA-based regimens. To estimate viral diversity from genotype-specific collections of sequences before DAA-treatment, we determined the pairwise amino acid distances between all sequences in respective sequence alignments downloaded from euHCVdb. Based on these sequence subsets, NS3h domain-interface residues of genotype 3 showed highest pairwise sequence diversity (widest breadth of viral population) with a maximum of 0.44 compared to genotype 1a (0.12), 1b (0.30) and 2 (0.17). Noticeable diversity is found in particular for the C-terminal helix α_18_ (residues 620 and 621) and helix α_14_ (residues 532 and 534) in NS3h. In contrast, residues of the protease-helicase allosteric site were found highly conserved throughout all genotypes (low sequence diversity). Physicochemical properties within the selected NS3h domain-interface residues were highly conserved and similar among all genotypes (Fig. 4). For variants deposited in the database but low- or none-replicating in the Con1 backbone, we could observe diverse second-site mutations throughout NS3 (no particular amino acid pattern identified; data not shown). Second-site mutations are also likely to occur outside NS3. A comprehensive analysis of second-site mutations, however, is out of scope of the present manuscript and will be subject of future investigations.

### Impact of NS3h domain-interface mutations on PI resistance

**Figure 5:**
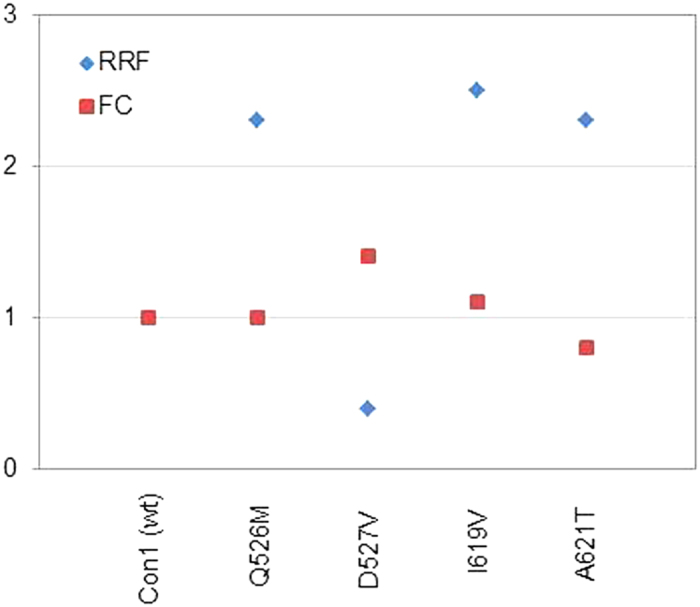
Resistance versus replicative fitness of natural helicase variants. Relative PI resistance expressed as the mean fold change (FC) in Con1 replicon IC_50_ values and relative replicative fitness (RRF) expressed as fold change relative to the Con1 wild type (y axis).

The impact of NS3h polymorphisms for PI resistance was investigated in four selected variants for the peptidomimetic PI telaprevir. The selection was made based on the replicative fitness and location of the polymorphism in the protein structure. IC_50_ values and fold changes (FC) were determined for the selected variants. Our data show no clinically significant effect for the selected NS3h variants on the inhibition of NS3p by telaprevir (Supplementary Fig. S2). An inverse correlation shows low resistance potential with high replicative fitness of variants (Fig. 5, Supplementary Table S1).

## Discussion

Diversity and genetic structure of a viral population are likely to determine their rapid adaptation and distinct pathogenesis. Prolonged replication of HCV in the liver environment during chronic infection result in numerous adaptive changes in the viral quasi-species that lead to an increase in viral fitness, thus suggesting that chronicity and advanced liver disease can render the virus less sensitive to antiviral treatment not only through accumulation of inhibitor resistance mutations in an expanded mutant spectrum but also through increasing replicative fitness^8^,^13^,^16^. RAVs may be associated with treatment outcome but virologic failure typically occurs only if other negative predictive factors are present at the same time. Viral variant (replicative) fitness is inextricably linked to RAV selection. Here we analyzed the natural variation in HCV dominant strains present among residues in the helicase domain, that in the NS3 closed conformation, are neighboring the protease natural substrate of proteolysis. We downloaded 652 HCV sequences of genotypes 1a, 1b, 2 and 3 from a public database, collected over years from geographically diverse sites. We cannot exclude the possibility that some of the variants we identified in these sets of sequences might represent variants that were present at low frequency in their source patient, or even unrecognized sequencing errors. However, because most variants were identified multiple times in the datasets it is likely that they represent true helicase variants present within the dominant quasi-species of the patients from whom these sequences were derived. Moreover, all database entries for variants characterized in the present work were created well before the first approval of DAAs against HCV, which precludes mutants tested in our present project from being archived resistance mutations against DAAs.

Fitness of a virus can be defined as its ‘relative ability to produce infectious progeny’^17^,^18^, which primarily relates to its replication capacity, and this is dependent on proper processing of the polyprotein by the NS3 protease. Yields of infectious virus generally correlate well with RNA replication capacity^9^,^18^. The mutants that we phenotypically characterized in our present work are located distant from the specific sites that were previously identified^18^ with greater impairment in their ability to produce infectious virus than predicted from reductions in RNA replication capacity. Some of the natural helicase variants that we tested were replication incompetent *in vitro*. The presence of these variants in the database but lack of replication in the Con1 replicon system that we applied in our study suggests that they are capable of functioning in an alternative sequence context. This highlights a limitation of the technical approach we have taken here, as second-site substitutions in the same strain might compensate for defects in replicative fitness. Although it might be preferable when investigating variants to engineer swaps of the complete NS3 sequence into the background of a replication-competent clone, this approach would frequently fail due to sequence incompatibilities between the donor and recipient viruses and the helicase specificity to recognize and interact with other components of the RNA synthesis complex from the same viral strain^19^. Our study shows that amino acid substitutions in the helicase domain of full-length NS3 can influence RNA replication with beneficial as well as detrimental impact on the variant replicative fitness when located close to the NS3 protease substrate of proteolysis but do not have direct impact on drug resistance against peptidomimetic PIs. We found modulatory residues primarily at two sites with most likely indirect impact on replicative fitness, (i) the NS3 domain-interface allosteric site and (ii) the helicase C-terminal helix α_18_. Importantly, amino acid polymorphisms derived from dominant variants and located in helix α_18_ had a positive effect on RNA replication, whereas allosteric site polymorphisms were found primarily with a distinct negative effect on replication. It is hence not surprising, that we found residues of the protease-helicase allosteric site highly conserved throughout all genotypes. In contrast to that, we found considerable sequence diversity at residues of the C-terminal helix α_18_ where amino acid substitutions had a strong and consistent positive effect on the variant replication capacity with genotype 3 showing significantly higher diversity than genotypes 1 and 2. Higher diversity in genotype 3 is also observed at helicase residue 532 that is neighboring helix α_18_ in the anti-parallel oriented helix α_14_. This seems an important characteristic that differentiates the most difficult-to-treat genotype 3 from other genotypes investigated in our study.

**Figure 6:**
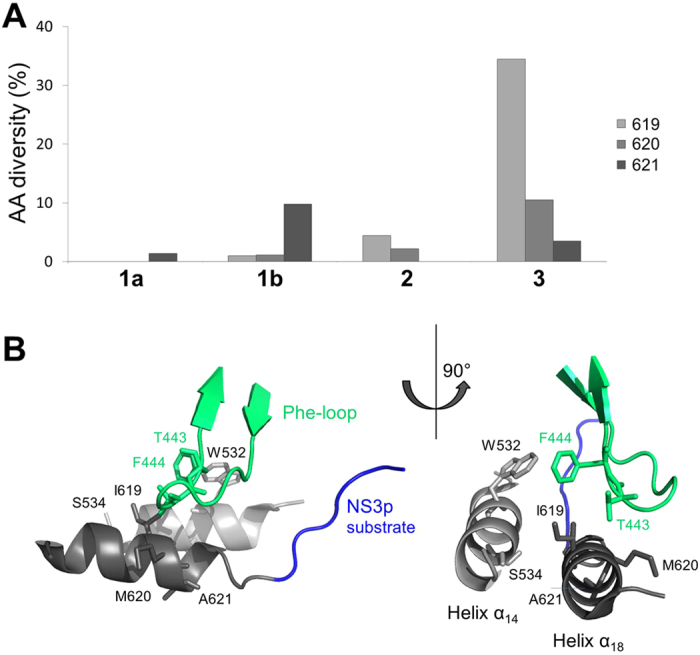
Amino acid diversity and protein structure context at helix α_18_. (**A**) Amino acid (AA) diversity given as percentage of amino acid mutations (y axis) observed at helix α_18_ residues 619, 620 and 621 in genotype 1a, 1b, 2 and 3 variants (x axis) from euHCVdb. (**B**) Detail of PDB structure 1CU1 showing helix α_18_ residues 619, 620 and 621 and neighboring residue side chains with potential impact for molecular changes in RNA replication in helicase variants.

Both NS3 domains are highly interdependent in that they modulate their respective biochemical activities with the domain interface taking a critical role in that interplay^20^,^21^. To investigate whether some of the residues and respective amino acid polymorphisms that we have analyzed in the helicase domain could be part of allosteric mechanisms related to the virus replication machinery, we were using the evolutionary history encoded within multiple sequence alignments. Correlated mutations are expected to reflect coordinated changes that maintain intramolecular coupling between residue pairs^22^. By using the co-evolutionary relationship between residue positions, we could identify a complex network of strongly coupled residue pairs within and between both NS3 domains with multiple connections via the domain interface. The network likely represents short- and long-range interactions within NS3 comprising the helicase motif IV (residue K371), involved in RNA replication and structure rearrangements, that is connected via the natural protease substrate site P_1_ (residue V630) with the protease amphipathic helix α_0_ (residue I18), required for efficient MAVS cleavage and RIG-I pathway control, which, in turn, is coupled to the helicase C-terminal helix α_18_ that we identified in our study with a regulatory role in RNA replication. Some additional functionally important sites were found associated to this closed I18-K371-V630 residue circuit, i.e. protease residue I170, involved in MAVS cleavage, helicase residue Y418, involved in intramolecular rearrangements and RNA interaction, as well as a subset of residues that connect helices α_14_ and α_18_ via the helicase Phe-loop. The respective residue side chains show potential molecular interactions that allow for conformational changes in variant structures that are likely to explain their modulatory impact on RNA replication (Fig. 6).

Partial overlap between the different sub-networks of co-evolving residues that we observed is consistent with coupling between different conformational states that happen during the formation of the viral replication complex. It has been previously demonstrated that amphipathic helix α_0_ of the NS3 protein associates with the ER membrane and positions the complex for the subsequent insertion of the N-terminus of NS4A into the membrane with subsequent rotation of the helicase domain away from the protease domain to form the replicase complex in NS3 extended conformation. A schematic representation of the conformational rearrangements and the protease and helicase relative position towards the ER membrane is given in Supplementary Fig. S3, showing the regulatory helices α_0_, α_14_ and α_18_ finally located in close structural proximity and oriented towards the phospholipid bilayer. The model initially proposed by Brass *et al.*^3^ suggests NS3 helix α_0_ and the transmembrane α-helix of NS4A involved in proper positioning of the serine protease active site on the ER membrane with important consequences for viral polyprotein processing and proteolytic targeting of host factors. The strict positioning of the protease active site with respect to the membrane confers a high degree of selectivity to potential cellular *trans*-cleavage substrates, such as MAVS^5^,^6^ or the membrane-associated peroxidase GPx8^23^. Our observations may add to this model in that they reflect pathways for information transfer between functional important sites in NS3 with helix α_18_ as a novel regulatory element. Noteworthy, we found in particular sites involved in RNA replication and proteolytic cleavage to co-evolve. The sequence diversity of genotype 3 at helix α_18_ is a most distinctive feature of this genotype that might allow for compensatory mechanisms in fitness deficits.

## Conclusions

Minor (modulatory) changes throughout the viral genome may add to RAV selection and may relate to virologic treatment failure. The structure element that we describe here is probably a novel functional site likely contributing to variant replicative fitness. High sequence diversity of i.e. genotype 3 at helix α_18_ is a remarkable feature of that genotype that suggests greater adaptive potential for escape from drug and immune pressure.

## Methods

### Sequence data

From the European HCV database (https://euhcvdb.ibcp.fr/euHCVdb/)^24^, we retrieved 218, 359, 46 and 29 worldwide NS3 sequences of strains from genotype 1a, 1b, 2 and 3 respectively. Sequences likely represent true variants present within the dominant quasi-species of the patients from whom these sequences were derived. Each amino acid reflects the most dominant amino acid at the respective position. The sequences were downloaded from the euHCVdb after filtering based on the following criteria: single protein with standard name ns3, confirmed genotype and published before 2011 prior to the approval of NS3 PIs or confirmed as derived from patients that either not have been treated with a PI or who were undergoing interferon therapy. To make certain that variants from the present paper are not archived resistance mutations rather than natural variants, we manually checked sequences for their submission date to the database. Identical sequences were removed. Multiple sequence alignments (MSAs) were computed using MUSCLE^25^ with default settings, manually corrected for sequencing errors, visualized and analyzed using the SEAVIEW alignment editor^26^. The Weizmann server^22^ was used for correlated mutation analysis based on genotype 1b NS3 full-length sequences (no residues clipped) and chi-square test (http://bip.weizmann.ac.il/correlated_mutations/). Sequence logos were computed using WebLogo (http://weblogo.berkeley.edu/logo.cgi)^27^ based on genotype-specific MSAs of NS3h domain-interface residues (see below, Structure data). Sequence logos were used to characterize the genotype-specific natural amino acid diversity within and between genotypes. Sequence diversity measures are computed using DIVEIN (http://indra.mullins.microbiol.washington.edu/DIVEIN/index.html)^28^. Given a collection of N sequences, the diversity is calculated as the mean distance between all sequences; d(*i,j*) is the genetic distance between sequences *i* and *j*:


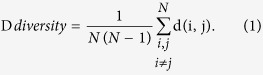


### Structure data

The crystal structure 1CU1^29^ from the Protein Databank RCSB PDB (www.rcsb.org)^30^ was used for *in silico* structure analysis. The structure contains the complete NS3 and NS4A sequence of genotype 1b (isolate BK) in a single polypeptide chain with NS3p and NS3h co-crystallized in closed conformation. The NS3p active site is occupied by the NS3h C-terminus as one product of NS3-mediated cleavage at the HCV polyprotein NS3-NS4A junction. Residues within a distance of ≤7.0 Å from the NS3p natural substrate of proteolysis were identified and denoted “NS3h domain-interface residues” with the cutoff based on previous publications. PyMOL and Chimera were used for protein structure analysis and visualization.

### Plasmids and *in vitro* RNA transcription

The plasmid pFKI_389_neo/NS3-3′/ET (kindly provided by Volker Lohmann and Ralf Bartenschlager, Heidelberg, Germany) contains HCV subgenomic replicon sequences derived from HCV genotype 1b and an upstream T7 promoter for *in vitro* RNA synthesis. The point mutations were generated with the QuikChange Lightning Site-Directed Mutagenesis Kit (Stratagene, La Jolla, CA, USA) and a set of primers given in Supplementary Table S2. The plasmid was linearized with ScaI and transcribed with the T7 RiboMAX Express Large Scale RNA Production System (Promega, Madison, WI, USA) according to the manufacturer’s instructions. Following treatment with RNase-free DNase to remove template DNA the resulting RNA transcripts were purified by RNA clean up with the RNeasy Mini Kit (Qiagen, Hilden, Germany). All plasmids and RNAs were checked for purity and integrity by standard procedures.

### Generation of HCV replicon cells

Huh7.5 cells were cultured in L-glutamine containing Dulbecco’s modified Eagle’s medium (DMEM; Gibco, Carlsbad, CA, USA) containing 10% fetal bovine serum (FBS; Sigma-Aldrich, St. Louis, MO, USA). Transfection of Huh7.5 cells with the *in vitro* transcribed subgenomic replicon RNA was performed using a standardized procedure (GenePulser, 975F, 270 V; Bio-Rad Laboratories GmbH; München, Germany). A wild-type sequence of pFKI_389_neo/NS3-3′/ET was used as positive control to compare with a replicon containing a lethal mutant (GDD^-^) as negative control. All mutants were tested at least in ten replicates.

### Four-day HCV replicon assay

Following transfection, HCV replicon cells were plated in duplicate or triplicate in 6 cm dishes at densities of 4–4.76 × 10^5^ cells in DMEM with 10% FBS. For telaprevir (VX-950, peptidomimetic PI) resistance analysis, 48 h later, the culture medium was replaced with DMEM + FBS containing DMSO as a control or the indicated concentrations of telaprevir (5mM stock in DMSO, Selleckchem, Houston, TX, USA). After an additional 48 h incubation, intracellular RNA was extracted with an RNeasy Mini kit (Qiagen). The level of HCV RNA was determined by two-step reverse transcription quantitative real-time PCR (RT-PCR) assay including cDNA synthesis (Prime Script RT Reagent Kit, Clontech, Mountain View, CA, USA) according to the manufacturer’s instructions and SYBR Green based RT-PCR (QuantiTect SYBR Green Kit, Qiagen, in 15μl reaction volume differing from the manufacturer’s instructions) with a pair of HCV-specific primers (HCV-S66: 5′-ACG CAG AAA GCG TCT AGC CAT-3′, HCV-A165: 5′-TAC TCA CCG GTT CCG CAG A-3′) on a StepOnePlus RT-PCR System (Applied Biosystems, Foster City, CA)^31^. Data were normalized to the expression of the reference gene GAPDH analyzed with the primer pair GAPDH-S: 5′-GAAGGTGAAGGTCGGAGTC-3′ and GAPDH-A: 5′-GAAGATGGTGATGGGATTTC-3′. Since GDD^−^ replicons do not generate additional HCV RNA, the GDD^−^ replicon was used to normalize data obtained with other replicons. Semi-quantitative HCV RNA replication was calculated using the DDCT method. Relative mRNA levels of the helicase variants were acquired and compared to wt, which was set to 1.

Relative replication efficiencies were displayed in relation to the wild type replicons replication capacity. Values were analyzed by student’s t-test for unpaired samples with a P value < 0.05 considered statistically significant. The IC_50_ was defined as the concentration of compound at which the HCV RNA level in the replicon cells was reduced by 50%, calculated by nonlinear regression analysis with GraphPad Prism.

## Additional Information

**How to cite this article**: Stross, C. *et al.* Natural HCV variants with increased replicative fitness due to NS3 helicase mutations in the C-terminal helix α_18_. *Sci. Rep.* **6**, 19526; doi: 10.1038/srep19526 (2016).

## Supplementary Material

Supplementary Information

## Figures and Tables

**Table 1:** Genotype/subtype-specific sequence diversity.

Pairwise based diversity		Max Median Min Mean	0.117 0 0 0.004	0.303 0 0 0.038	0.172 0 0 0.017	0.439 0.051 0 0.067
Residue	Wild type	Variant	Genotype/Subtype			
			1a	1b	2	3
438	F	L	–	1	–	–
488	S	T	–	1	1 (2.2%)	–
524	V	F	–	2	–	–
		I		2	–	
525	C	W	–	2	–	–
		H	–	–	1 (2.2%)	–
526	Q	M	–	1	–	–
		H	1	–	–	–
527	D	V	–	1	–	–
528	H	Y	–	1	–	–
529	L	–	–	–	–	–
530	E (1a, 1b, 2) D (3)	G	1	–	–	–
		E	–	–	–	2 (6.9%)
531	F	L	–	–	–	1 (3.5%)
532	W	S	–	2	–	–
534	G (1a)S (1b, 3) A (2)	G	1	74 (20.6%)	2 (4.4%)	1 (3.5%)
		A	1	1	–	–
		D		1	–	–
		R	–	–	–	1 (3.5%)
619	I	V	–	3	2 (4.4%)	7 (24.1%)
		L	–	–	–	2 (6.9%)
		N	–	–	–	1 (3.5%)
620	M (1a, 2, 3)A (1b)	T	–	4 (1.1%)		1 (3.5%)
		G	–	–	1 (2.2%)	–
		A	–	–	–	1 (3.5%)
		K	–	–	–	1 (3.5%)
621	T (1a, 2) A (1b, 3)	T	3 (1.4%)	35 (9.8%)		1 (3.5%)
622	C	S	–	1	–	–
623	M	–	–	–	–	–
624	S (1a, 1b, 3) Q (2)	A	1	10 (2.8%)	–	1 (3.5%)
		W	–	1	–	

Variants at helicase residues are summarized together with their respective absolute number and relative abundance (in %) from the euHCVdb. Frequencies are only indicated when ≥1%. Column two indicates the wild-type residue (if the wild type residue differs between genotypes, the respective genotype/subtype is given in brackets).

**Table 2:** Coupled residue pairs in full-length NS3.

Residue i	Residue j	df	χ^2^	P value
I18	K371	3	37.763	0.0000
I18	Y618	5	30.335	0.0000
I18	V630	3	84.483	0.0000
S42	Y56	3	22.379	0.0001
V51	I170	3	19.836	0.0002
S122	H246	7	24.962	0.0008
I170	A263	5	23.998	0.0002
I170	V630	3	19.406	0.0002
S299	Y418	3	96.082	0.0000
I303	D555	3	53.138	0.0000
K371	V630	3	99.246	0.0000
Y418	Y618	5	21.235	0.0007
T510	Y618	5	31.807	0.0000
T510	T621	3	61.427	0.0000

Pairs of positions (i,j) in full-length NS3 of genotype 1b with correlated mutations found to be statistically significant. Results for any two positions i,j are considered significant if P < 0.05 unless: (1) any of the N_n,Ex_ is less than one, or (2) if more than 20% of the N_n,EX_ are less than five. If P ≤ 0.001 the results are considered significant even if condition (2) is not met. N_Ex_ is the expected number of sequences that contain amino acid X at position i and amino acid Y at position j. A difference between the observed number of sequences that contain amino acid X at position i and amino acid Y at position j, N_Obs_, and N_Ex_ may indicate residue coupling. Statistical significance for that is estimated using the χ^2^ goodness-of-fit test. The significance of the χ^2^ values depends on the number of degrees of freedom (df).
