# Supplementary material for: Natural HCV variants with increased replicative fitness due to NS3 helicase mutations in the C-terminal helix α18
Source: Sci Rep. 2016 Jan 20;6:19526. doi: 10.1038/srep19526 (PMC4726148; doi:10.1038/srep19526)
Supplement: Supplementary Information [file srep19526-s1.docx]

**Supplementary Information**

**Natural HCV variants with increased replicative fitness due to NS3 helicase mutations in the C-terminal helix α_18_**

Claudia Stross^1^, Tetsuro Shimakami^2^, Katrin Haselow^1^, Monazza Q. Ahmad^1^, Stefan Zeuzem^1^, Christian M. Lange^1^, Christoph Welsch^1*^

^1^Department of Internal Medicine 1, Goethe University Hospital Frankfurt, 60590 Frankfurt a.M., Germany ^2^Department of Gastroenterology, Kanazawa University Hospital, Kanazawa, Japan

*Corresponding author: Department of Internal Medicine 1, Goethe University Hospital Frankfurt, 60590 Frankfurt a.M., Germany, email: christoph.welsch@kgu.de

**Supplementary Figure Legends.**

**Supplementary Figure S1. Protease-helicase complex structure.** PDB structure 1CU1 (ribbon model; NS3h in dark green, NS3p in light green, NS4A in pale green) and NS3h amino acid residues within close structure proximity (≤ 7.0 Å) to the natural substrate of NS3p-mediated proteolysis (green stick model in the domain interface) given as CPK models (with amino acid polymorphisms in dominant strains from euHCVdb in red, without polymorphisms in orange).

**Supplementary Figure S2. In-vitro IC_50_ determination of natural helicase variants.** Dose-dependent reduction of HCV RNA by the protease inhibitor telaprevir assessed in replicon cells harboring wild-type Con1 and selected helicase variants. The replicon cells were incubated with various concentrations of telaprevir for 48h. At the end of the incubation period, total RNA was extracted, and the levels of HCV RNA remaining were determined by two-step reverse transcription quantitative real-time PCR (RT-PCR), normalized to that of the wild-type Con1 RNA. Relative replicative fitness is given on the y axis, the respective telaprevir concentration is given on the x axis. IC_50_ is determined with formula IC_50_ = ( 1 + [S] / *K_m_*) ∙ *K_i_*  (Equation 2) in GraphPad Prism.

**Supplementary Figure S3. Protease-helicase conformational rearrangements.** Schematic representation of the NS3 protease and helicase relative position towards the ER membrane according to a model by Brass et al. [3] showing the regulatory helices α_0_, α_14_ and α_18_ finally located in close structural proximity and oriented towards the phospholipid bilayer. Coupled residue-pairs are indicated as nodes and color coded (see implemented legend in figure).

**Supplementary Figures.**

**Supplementary Figure S1.**

**
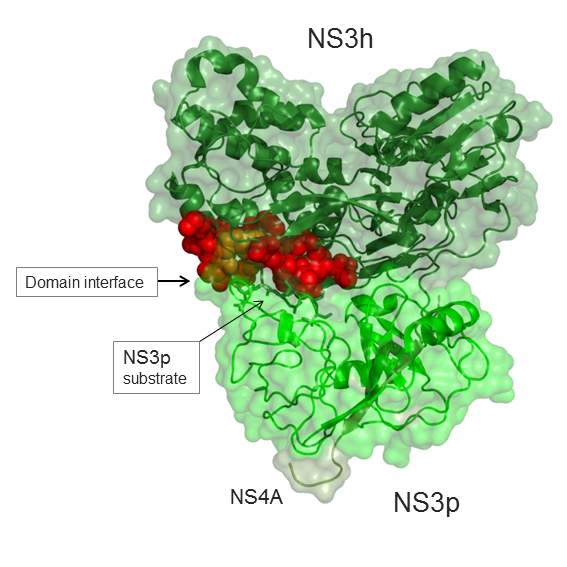
**

**Supplementary Figure S2.**

**
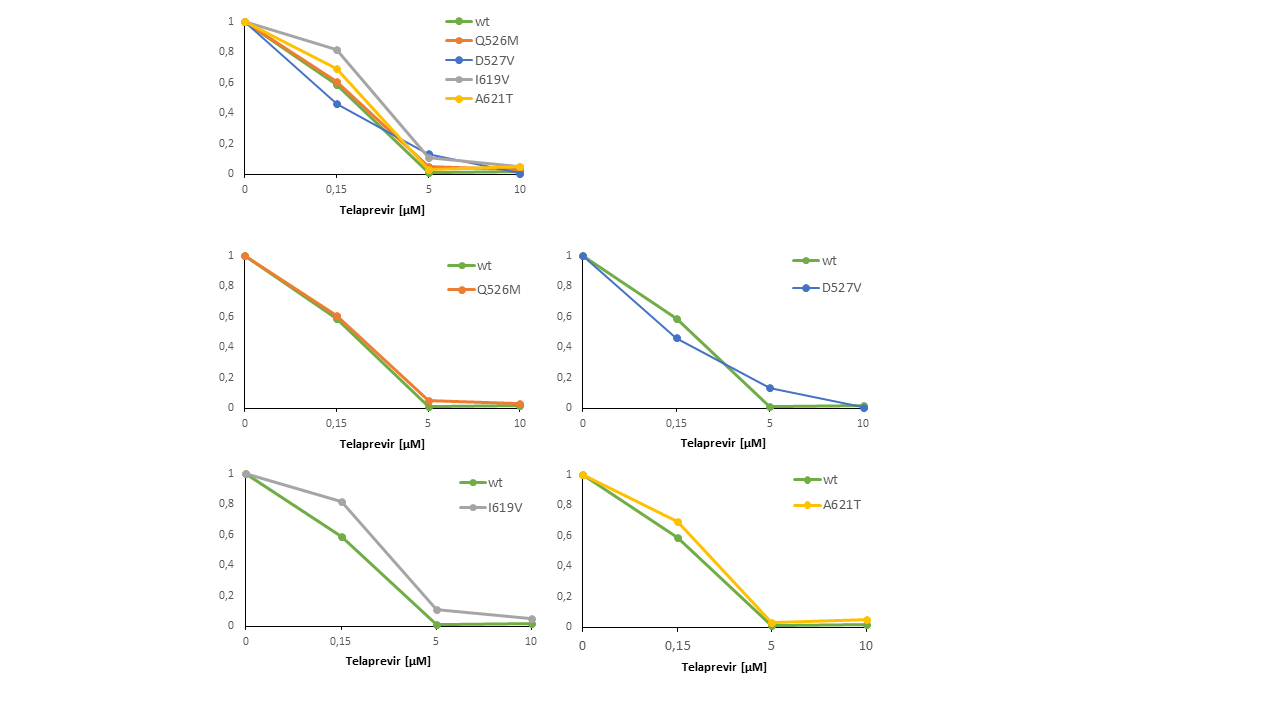
**

**Supplementary Figure S3.**

**
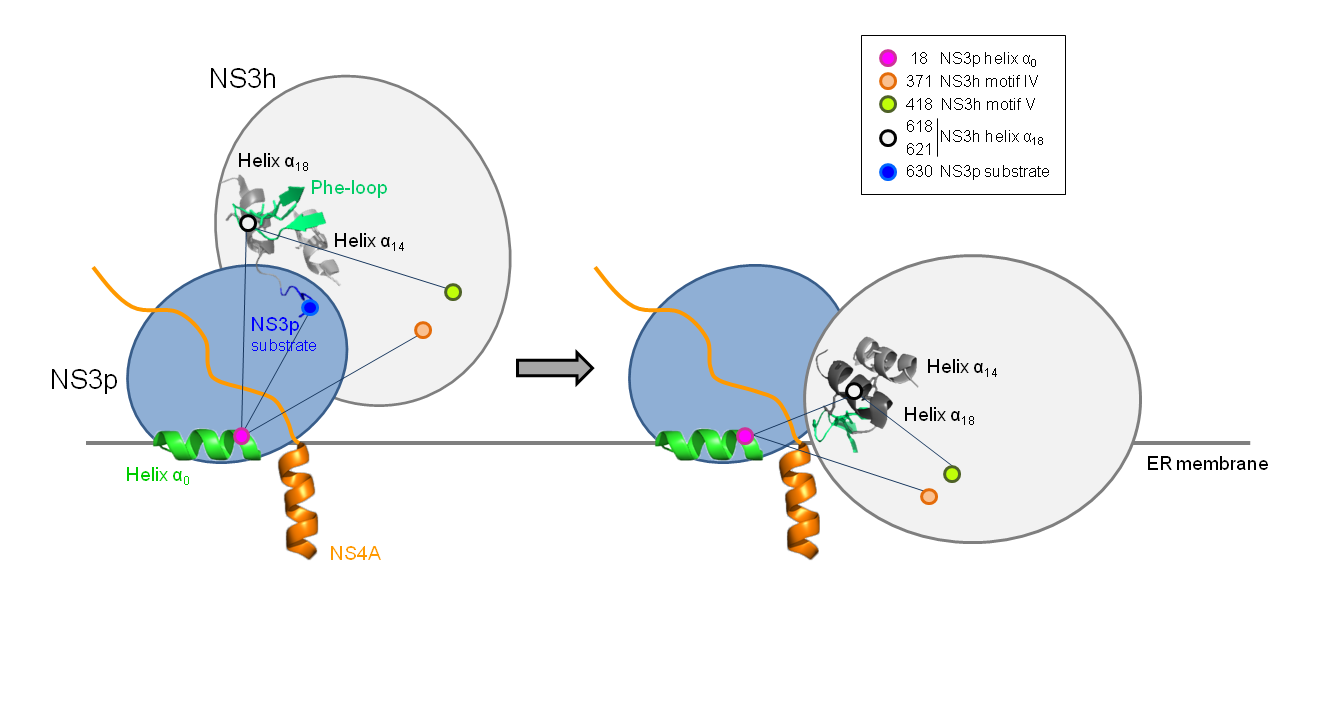
**

**Supplementary Tables.**

**Supplementary Table S1. Replicative fitness and drug resistance of natural helicase variants *in vitro*.**

|  | **Replicative fitness** | | | **Drug resistance** | | | |
| --- | --- | --- | --- | --- | --- | --- | --- |
| **Mutant** | **RRF** | **SD** | **P value** | **IC_50_ (nM)** | **FC** | **SD** | **P value** |
| wt (Con1) | 1 | 0.0 | - | 156 | 1.0 | - | - |
| V524F | 0.00 | 0.0 | 0.00 | nd | nd | nd | nd |
| V524I | 0.24 | 0.13 | 0.00 | nd | nd | nd | nd |
| C525W | 0.02 | 0.18 | 0.00 | nd | nd | nd | nd |
| Q526M | 1.88 | 1.28 | 0.01 | 186 | 1.2 | 0.3 | 0.23 |
| D527V | 0.43 | 0.21 | 0.01 | 128 | 0.8 | nd | nd |
| H528Y | 0.03 | 0.02 | 0.00 | nd | nd | nd | nd |
| W532S | 0.01 | 0.13 | 0.00 | nd | nd | nd | nd |
| S534G | 0.76 | 0.58 | 0.12 | nd | nd | nd | nd |
| F438L | 0.39 | 0.20 | 0.00 | nd | nd | nd | nd |
| I619V | 1.97 | 1.18 | 0.00 | 263 | 1.7 | 0.4 | 0.13 |
| M620T | 0.88 | 0.44 | 0.28 | nd | nd | nd | nd |
| A621T | 1.40 | 0.72 | 0.07 | 159 | 1.0 | 0.2 | 0.45 |
| S624A | 1.16 | 0.67 | 0.28 | nd | nd | nd | nd |

On the left: Relative replicative fitness in relation to wild-type (wt) replicon; relative replicative fitness (RRF) and standard deviation (SD). On the right: Enzymatic drug resistance data for the peptidomimetic PI telaprevir compared to wt; IC_50_ values, fold changes (FC) and standard deviation (SD). Values analyzed by student’s t-test for unpaired samples with a P value < 0.05 considered statistically significant.

**Supplementary Table S2. Primers used for NS3h site-directed mutagenesis.**

|  | **Primer sense** | **Primer antisense** |
| --- | --- | --- |
| **V524F** | CACACCAGGGTTGCCCTTCTGCCAGGACCATCTGG | CCAGATGGTCCTGGCAGAAGGGCAACCCTGGTGTG |
| **V524I** | CACACCAGGGTTGCCCATCTGCCAGGACCATCTGG | CCAGATGGTCCTGGCAGATGGGCAACCCTGGTGTG |
| **C525W** | CCAGGGTTGCCCGTCTGGCAGGACCATCTGGAGTTC | GAACTCCAGATGGTCCTGCCAGACGGGCAACCCTGG |
| **W532S** | GGACCATCTGGAGTTCTCGGAGAGCGTCTTTACAGGCC | GGCCTGTAAAGACGCTCTCCGAGAACTCCAGATGGTCC |
| **S534G** | CATCTGGAGTTCTGGGAGGGCGTCTTTACAGGCCTCAC | GTGAGGCCTGTAAAGACGCCCTCCCAGAACTCCAGATG |
| **I619V** | CACCCCATAACCAAATACGTCATGGCATGCATGTCGGCTGAC | GTCAGCCGACATGCATGCCATGACGTATTTGGTTATGGGGTG |
| **M620T** | CCATAACCAAATACATCACGGCATGCATGTCGGCTGACCTGGAG | CTCCAGGTCAGCCGACATGCATGCCGTGATGTATTTGGTTATGG |
| **A621T** | CCCATAACCAAATACATCATGACATGCATGTCGGCTGACCTGGAGG | CCTCCAGGTCAGCCGACATGCATGTCATGATGTATTTGGTTATGGG |
| **S624A** | CATCATGGCATGCATGGCGGCTGACCTGGAGG | CCTCCAGGTCAGCCGCCATGCATGCCATGATG |
| **F438L** | CCAGACAGTCGACTTAAGCCTGGACCCGACC | GGTCGGGTCCAGGCTTAAGTCGACTGTCTGG |
| **D527V** | CCGTCTGCCAGGTCCATCTGGAGTTC | GAACTCCAGATGGACCTGGCAGACGG |
| **H528Y** | CGTCTGCCAGGACTATCTGGAGTTCTGG | CCAGAACTCCAGATAGTCCTGGCAGACG |
| **Q526M** | GTTGCCCGTCTGCATGGACCATCTGGAG | CTCCAGATGGTCCATGCAGACGGGCAAC |
